# Dialysis enrollment patterns in Guatemala: evidence of the chronic kidney disease of non-traditional causes epidemic in Mesoamerica

**DOI:** 10.1186/s12882-015-0049-x

**Published:** 2015-04-14

**Authors:** Timothy S Laux, Joaquin Barnoya, Douglas R Guerrero, Marcos Rothstein

**Affiliations:** Barnes-Jewish Hospital Department of Internal Medicine, St. Louis, MO USA; Department of Medicine, Division of Medical Education, Washington University School of Medicine, 660 South Euclid Avenue, Box 8121, St Louis, MO 63110 USA; Washington University in St Louis Division of Public Health Sciences, 660 S. Euclid Avenue, Campus Box 8100, St. Louis, MO 63110 USA; Unidad Nacional de Atención al Enfermo Renal Crónico, 9a. Avenida 3-40 Zona 1, Ciudad de Guatemala, 01001 Guatemala Centro América; Division of Renal Diseases, Washington University in St. Louis, St. Louis, MO USA; Washington University School of Medicine, Division of Renal Diseases, 660 South Euclid Avenue, Campus Box 8126, St. Louis, MO 63110 USA

**Keywords:** Central America, Chronic kidney disease, Dialysis, Guatemala, Human development index, Literacy

## Abstract

**Background:**

In western Nicaragua and El Salvador, chronic kidney disease (CKD) is highly prevalent and generally affects young, male, agricultural (usually sugar cane) workers without the established CKD risk factors. It is yet unknown if the prevalence of this CKD of Non-Traditional causes (CKDnT) extends to the northernmost Central American country, Guatemala. Therefore, we sought to compare dialysis enrollment rates by region, municipality, sex, daily temperature, and agricultural production in Guatemala and assess if there is a similar CKDnT distribution pattern as in Nicaragua and El Salvador.

**Methods:**

The National Center for Chronic Kidney Disease Treatment (Unidad Nacional de Atención al Enfermo Renal Crónico) is the largest provider of dialysis in Guatemala. We used population, Human Development Index, literacy, and agricultural databases to assess the geographic, economic, and educational correlations with the National Center for Chronic Kidney Disease Treatment’s hemodialysis and peritoneal dialysis enrollment database. Enrollment rates (per 100 000) inhabitants were compared by region and mapped for comparison to regional agricultural and daytime temperature data. The distribution of men and women enrolled in dialysis were compared by region using Fisher’s exact tests. Spearman’s rank correlation coefficients were calculated.

**Results:**

Dialysis enrollment is higher in the Southwest compared to the rest of the country where enrollees are more likely (p < 0.01) to be male (57.8%) compared to the rest of the country (49.3%). Dialysis enrollment positively correlates with Human Development Index and literacy rates. These correlations are weaker in the agricultural regions (predominantly sugar cane) of Southwest Guatemala.

**Conclusions:**

In Guatemala, CKDnT incidence may have a similar geographic distribution as Nicaragua and El Salvador (higher in the high temperature and sugar cane growing regions). Therefore, it is likely that the CKNnT epidemic extends throughout the Mesoamerican region.

## Background

Chronic kidney disease (CKD) prevalence is growing worldwide [1]. While CKD is most commonly caused by diabetes and hypertension, local epidemics of CKD have been described, some of unknown origin [1,2]. Locations affected include India [3], Sri Lanka [4,5], Egypt [6], and Central America [7-9]. In western Nicaragua, CKD is five times more common among men than women and is the leading cause of death (95 deaths per 100 000 residents) among men [10]. In the city of Chichigalpa, Nicaragua, 46% of all male deaths from 2002–2012 were attributed to CKD [11]. Similarly, among men in in El Salvador, it is the second leading cause of death [12,13] and, in 2009 and 2011, was the most common cause of in-hospital death [14,15]. Causes for this high prevalence remain to be determined, hence the terms CKD of Non-Traditional Causes (CKDnT), of Unknown Origin (CKDu) and Mesoamerican Nephropathy (MeN) [7,10,16,17]. Multiple hypotheses have been proposed, including work related heat stress and recurrent dehydration (e.g., linked to intense agricultural work like sugar cane cutting) [2,7,9,10,12,14-27], agro-chemicals exposure [2,7,9,10,12,14,15,20,24,25,28], nephrotoxic medication use (e.g. non-steroidal anti-inflammatory drugs) [7,10,12,14-17,20,25], low birth weight [7,10], and fructokinase mediated injury secondary to sweetened beverage consumption or sugar cane chewing [7,16,20,24,26,27].

Guatemala has some of the highest rates of CKD and renal failure mortality seen in the Americas [29]. Like its Mesoamerican neighbors, Nicaragua and El Salvador, Guatemala shares many of the proposed risk factors for CKDnT. However, it is unknown if CKD (and, possibly, CKDnT) rates in Guatemala are higher in low-altitude agricultural regions in the Southwest as has been described in Nicaragua and El Salvador [12,17,21-23,30].

Therefore, we sought to compare dialysis enrollment rates by region, municipality, and sex and assess if there is a difference in the Southwest by economic development, literacy level, daily temperatures, and agricultural production.

## Methods

The National Center for Chronic Kidney Disease Treatment (Unidad Nacional de Atención al Enfermo Renal Crónico, UNAERC) maintains a database of all individuals enrolled in dialysis since 2008 (n = 3 105). This database includes patient’s sex, dialysis type (hemodialysis or peritoneal [PD]), and home address. Using projected 2012 census data [31], UNAERC calculated the dialysis enrollment rate per 100 000 inhabitants of each department and municipality. As of March 2013, data from patients enrolled in dialysis in 258 of Guatemala’s 332 municipalities (78%) in all 22 departments (100%) was available. In the Southwest, data was missing from 9 of 102 municipalities (8.8%), while data was missing from 65 of 230 municipalities (28.3%) in the rest of the country.

Dialysis enrollment rates were mapped using ArcGIS software (Esri, Redlands, California, United States). To determine if there is a geographic correlation between enrollment rates and sugar cane growing regions, dialysis enrollment rates were superimposed onto maps of sugar cane plantations. While sugar cane production is not the only crop in Guatemala, it is the predominant and most economically important in the Southwest departments (n = 5). Additionally, the high temperatures and flat land needed for these plantations are only available in the Southwest. This same mapping was also performed for dialysis enrollment and average daytime harvest temperatures in the Southwest. Both the sugar cane plantations and weather data are available from and used with the permission of the Guatemala Sugar Cane Research and Training Center (Centro Guatemalteco de Investigación y Capacitación de la Caña de Azúcar, Cengicaña).

Referral rates by sex were compared between the Southwest departments (n = 5) and the rest of the country (n = 17). Analyses of referral rate patterns were performed using Fisher’s exact tests. Correlations between dialysis enrollment rates and Human Development Index (HDI) and literacy rates for each municipality [32] were estimated using Spearman’s rank (i.e., Spearman’s rho [r_s_]). HDI is a composite statistic which includes data concerning life expectancy, educational attainment, and wealth [33]. The correlation coefficients were compared between the entire nation (22 departments), Southwest Guatemala (5 departments), and the rest of Guatemala (17 departments).

As this study involved publicly available data with no patient identifiers, it was deemed exempt from Institutional Review Board or Ethics Committee approval. Permission was obtained from UNAERC to complete this study.

## Results

**Figure 1:**
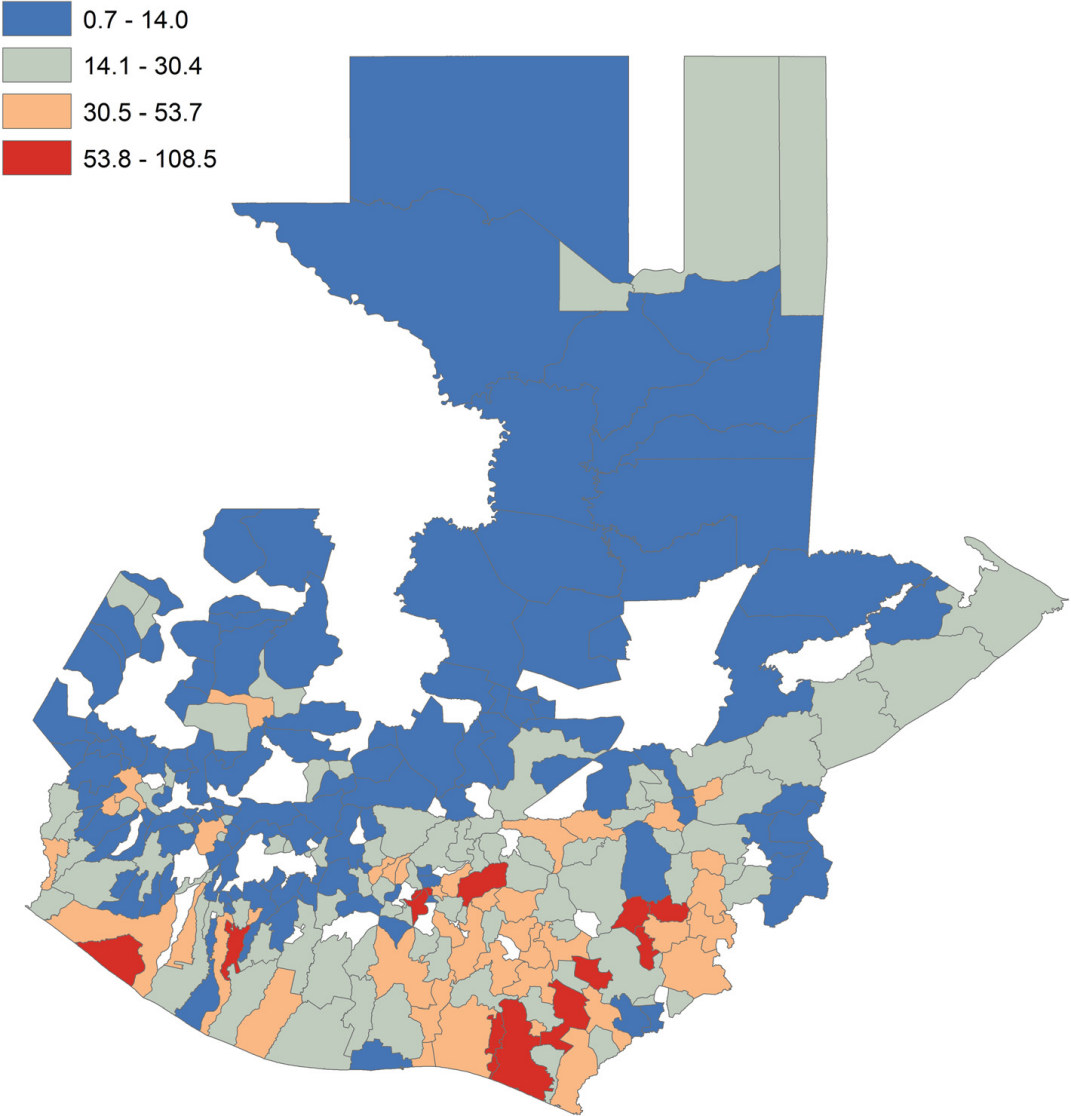
Dialysis enrollment rates (per 100 000 Residents) by Municipality in Guatemala. Enrollment rates (per 100 000 residents) are shown at the municipal level for all of Guatemala. Municipalities shown in white do not have data.

**Figure 2:**
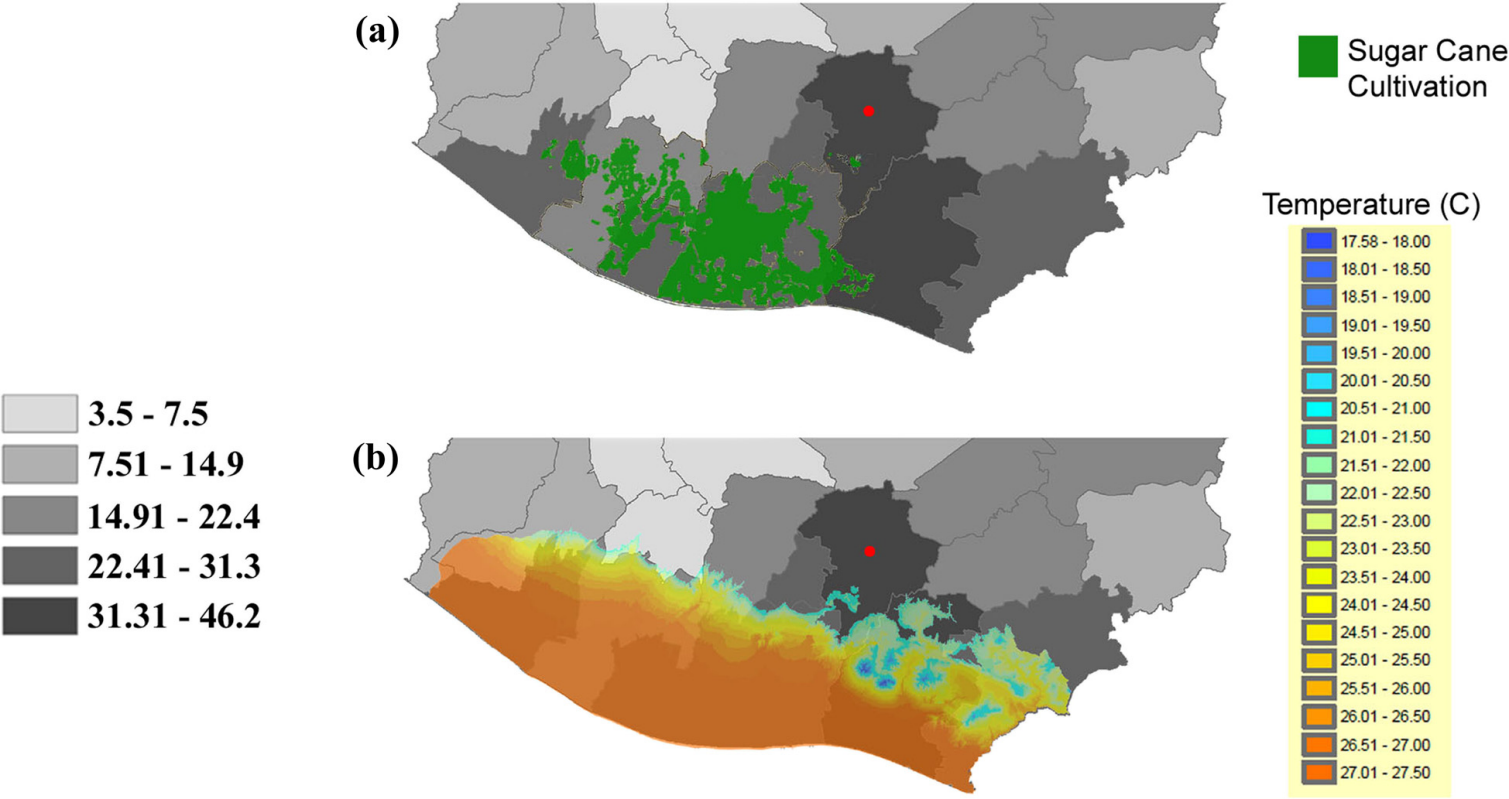
Dialysis enrollment rates (per 100 000 residents) compared with agricultural and temperature data in Southwest Guatemala. Enrollment rates (per 100 000 residents) are shown at the departmental level for Southwest Guatemala. Part **(a)** demonstrates location of known sugar cane plantations (data available from Cengicaña) and part **(b)** shows average daytime temperature data during the sugar cane harvest season (also available from Cengicaña) superimposed on the same departmental dialysis enrollment rates. The capital department is shown with a red dot in parts **(a)** and **(b)**.

Rates per 100 000 residents of individuals enrolled in dialysis were highest in the Southwest, shown at the municipal level (Figure 1). Locations of sugar cane plantations (Figure 2a) and higher daytime temperatures (Figure 2b) during the harvest season (November to April) closely overlap with dialysis enrollment rates.

Sex distribution was different between the Southwest departments and the rest of the country (Table 1) for all dialysis (p < 0.01) and PD (p < 0.01), but not hemodialysis (p = 0.14). In the Southwest, 57.8% enrollees were male (57.3% of hemodialysis and 57.9% of PD) compared to 49.3% in the other departments (50.3% and 48.9%, respectively). PD is the most common dialysis modality in all of Guatemala (Table 2), but is more common in the Southwest (79.9%) than the rest of the country (75.7%, p < 0.01).

**Table 1:** **Enrollment (by Sex) in all, peritoneal, and hemodialysis by region in Guatemala**

	**All dialysis**			**Hemodialysis**			**Peritoneal dialysis**		
**Location**	**Men (n, %)**	**Women (n, %)**	**p**	**Men (n, %)**	**Women (n, %)**	**p**	**Men (n, %)**	**Women (n, %)**	**p**
**All Guatemala**	1 591 (51.2%)	1 514 (48.8%)	-----	389 (51.7%)	364 (48.3%)	-----	1 202 (51.1%)	1 150 (48.9%)	-----
**Southwest (n = 5)**	412 (57.8%)	301 (42.2%)	< 0.01	82 (57.3%)	61 (42.7%)	0.14	330 (57.9%)	240 (42.1%)	< 0.01
**Other** **(n = 17)**	1 179 (49.3%)	1 213 (50.7%)		307 (50.3%)	303 (49.7%)		872 (48.9%)	910 (51.1%)

**Table 2:** **Enrollment in dialysis type by region**

**Location**	**Hemodialysis (n, %)**	**Peritoneal Dialysis (n, %)**	**p-value**
**All Guatemala (n = 3 105)**	753 (24.3%)	2 352 (75.7%)	--
**Southwest (n =713)**	143 (20.1%)	570 (79.9%)	< 0.01
**Other (n = 2 392)**	610 (25.5%)	1 782 (74.5%)	

**Figure 3:**
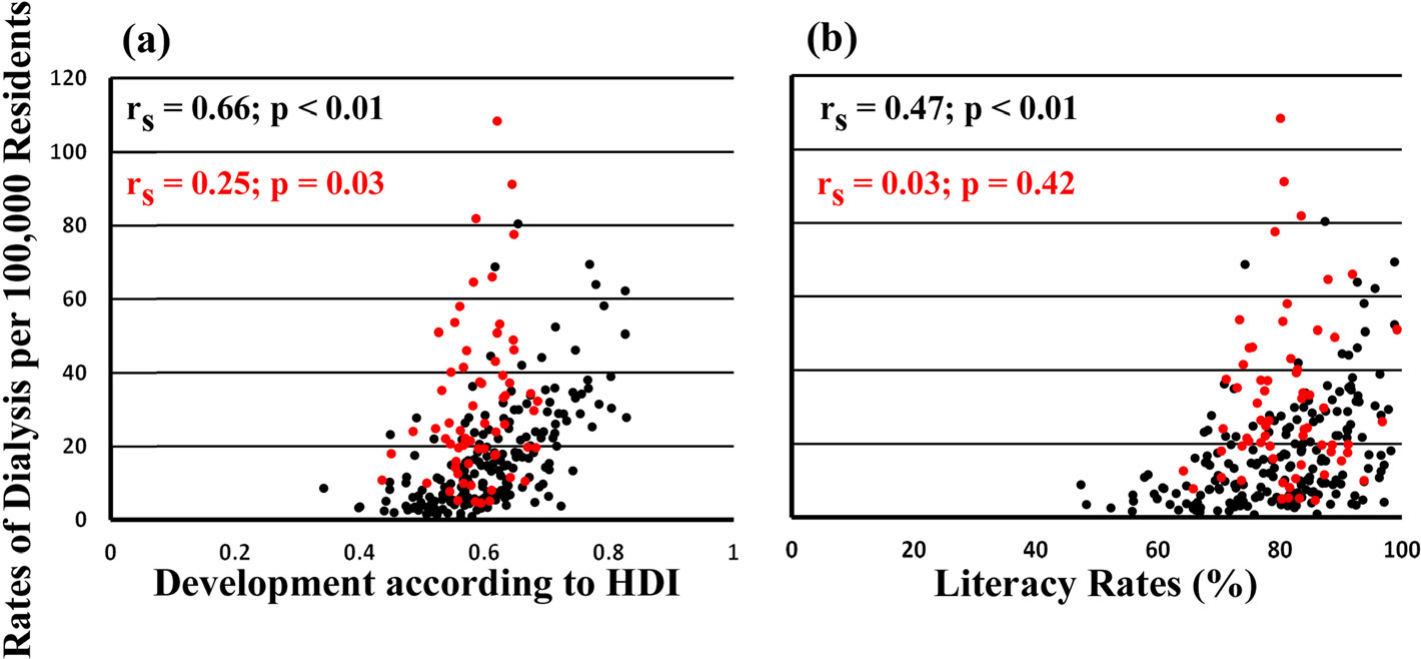
Dialysis Enrollment Rates (per 100 000 Residents) by Human Development Index and Literacy Rates in Guatemala. **(a)** Scatter plot of municipal dialysis enrollment per 100 000 residents by Human Development Index (HDI) for the 17 departments located outside of Southwest Guatemala (in black) and Southwestern Guatemala (in red). **(b)** Scatter plot of municipal dialysis enrollment per 100 000 residents by literacy rates for the 17 departments located outside of Southwest Guatemala (in black) and Southwestern Guatemala (in red). Spearman’s correlation coefficients (rs) and p-values are shown for each region and are color coded in the same manner.

HDI and literacy rates increased at the national level as municipal dialysis enrollment rates increased (For HDI (range 0.34-0.83): r_s_ = 0.50, p < 0.01; for Literacy: r_s_ = 0.37, p < 0.01). The relationship between HDI / literacy rates and dialysis enrollment rates for both Southwest Guatemala (shown in red) and the 17 other departments (shown in black) appear in Figure 3. A stronger positive correlation was found between HDI and dialysis enrollment rates when only the municipalities in the 17 other departments were analyzed (Figure 3a, in black, HDI range 0.34-0.83, r_s_ = 0.66, p < 0.01) as compared to the weaker correlation in the Southwest departments (Figure 3a, in red, HDI range 0.40-0.75, r_s_ = 0.25, p = 0.03). Municipal literacy and dialysis enrollment rates were also strongly correlated in the 17 other departments (Figure 3b, in black, r_s_ = 0.47, p < 0.01). No correlation between municipal literacy rates and dialysis enrollment rates was seen in the Southwest (Figure 3b, in red, r_s_ = 0.03, p = 0.42).

Regarding dialysis type, a positive correlation between municipal dialysis enrollment rates and HDI was found for hemodialysis and PD (Table 3) that persisted when stratified by sex. This correlation, however, was weakest in Southwest Guatemala, especially among men enrolled in PD. The same pattern was seen when comparing municipal hemodialysis and PD enrollment rates and literacy rates (Table 3). The smallest correlation was again seen among men enrolled in PD in the Southwest.

**Table 3:** **Correlations between human development index (HDI) and literacy rate by region, dialysis type and sex**

**Indicator**	**Location**	**HD – All (r**_**s**_ **)**	**HD – Men (r**_**s**_ **)**	**HD – Women (r**_**s**_ **)**	**PD – All (r**_**s**_ **)**	**PD – Men (r**_**s**_ **)**	**PD – Women (r**_**s**_ **)**
**Human Development Index**	**All Guatemala (n = 258 municipalities in 22 departments)**	r_s_ = 0.39^	r_s_ = 0.28^	r_s_ = 0.34^	r_s_ = 0.47^	r_s_ = 0.42^	r_s_ = 0.35^
	**Southwest Guatemala (n = 64 municipalities in 5 departments)**	r_s_ = 0.47^	r_s_ = 0.25*	r_s_ = 0.23*	r_s_ = 0.23*	r_s_ = 0.16	r_s_ = 0.21*
	**Other Guatemala (n = 194 municipalities in 17 departments)**	r_s_ = 0.46^	r_s_ = 0.38^	r_s_ = 0.43^	r_s_ = 0.61^	r_s_ = 0.54^	r_s_ = 0.45^
**Literacy Rates**	**All Guatemala (n = 258 municipalities in 22 departments)**	r_s_ = 0.25^	r_s_ = 0.17^	r_s_ = 0.20^	r_s_ = 0.35^	r_s_ = 0.29^	r_s_ = 0.25^
	**Southwest Guatemala (n = 64 municipalities in 5 departments)**	r_s_ = 0.31*	r_s_ = 0.11	r_s_ = 0.14	r_s_ = −0.03	r_s_ = −0.05	r_s_ = 0.04
	**Other Guatemala (n = 194 municipalities in 17 departments)**	r_s_ = 0.26^	r_s_ = 0.21^	r_s_ = 0.22^	r_s_ = 0.45^	r_s_ = 0.39^	r_s_ = 0.31^

*p = 0.01-0.05.

^ p < 0.01.

## Discussion

According to our findings, dialysis enrollment rates in Guatemala are higher in the Southwest. There, individuals are both more likely to be male and more likely to be enrolled in PD. These findings are particularly interesting because the Southwest is generally poorer than the rest of the country. It seems plausible to suggest that accessing dialysis would therefore be more difficult in these municipalities. The trends we noted in Guatemala closely parallel the pattern seen at the community level elsewhere in Central America, where the highest prevalence of CKD is seen in coastal Pacific communities among men. In Central America, the coastal Pacific regions are the hot, low-altitude environments in which sugar cane is grown.

The 3:2 male:female ratio of dialysis enrollees in Southwest Guatemala is smaller than the 3–4:1 ratios of CKD prevalence and mortality documented elsewhere in Central America [7]. These differences are difficult to interpret as our unit of measure (dialysis enrollment rates by municipality and department from 2008–13) is different than any previously published. We do not believe that gender roles or occupational patterns in Southwest Guatemala are different from regions with higher documented ratios.

Our findings are in agreement with unpublished 2013 CKD mortality data from the Guatemalan Ministry of Health (personal communication). While the Ministry of Health data is organized by geographic areas-of-health, four of the top five CKD mortality rates are found in areas-of-health in the Southwest, the region with highest dialysis enrollment rates. In publicly available data from the Pan American Health Organization for 2005–08 [29], CKD and renal failure mortality in Guatemala has consistently been in the top 10 among reporting nations in Latin America.

The positive correlations between municipal dialysis enrollment rates, HDI’s and literacy rates argue that access to dialysis is associated with wealth and education in most of Guatemala. The region where these correlations are weaker (or non-existent) is the Southwest, especially among men enrolled in PD. This could be explained by higher rates of End Stage Renal Disease among low-literacy individuals living in poorer municipalities, who are generally enrolled in PD due to a lack of access to Guatemala’s hemodialysis infrastructure. We hypothesize that many of these individuals are agricultural workers [34], most of whom likely work in sugar cane plantations during the harvest season. These individuals’ kidney failure is most likely secondary to CKDnT. This explanation, however, is speculative.

UNAERC classifies patients by municipality of residence and not municipality of work. Many individuals with End Stage Renal Disease often move to the capital department to better access dialysis. This referral bias may explain why the capital department (shown in Figure 2a and b with the red dots) appears to have higher enrollment rates despite being located outside Southwest Guatemala.

Our data has strengths and limitations. To the best of our knowledge, this is the first time dialysis enrollment rates in Guatemala (or any Mesoamerican country) have been correlated with an economic (HDI) or educational (literacy rates) indicator and only the second time maps of agricultural activity and regional temperature have been used to analyze CKDnT [35].

However, individuals enroll in dialysis through multiple institutions in Guatemala and even though UNAERC is the largest dialysis provider to the public sector (including agricultural workers), those receiving treatment elsewhere are not included in our database. UNAERC’s database is also more complete for Southwest Guatemala (91.2% of municipalities reporting), though it is still quite complete for the rest of Guatemala (71.7% of municipalities reporting). It is unclear why this occurred and if this affects the overall dialysis enrollment trends in either region. In addition, UNAERC’s database is limited and lacks data on kidney failure causes, occupational history, patient age, or age at enrollment. Finally, dialysis enrollment was used as a proxy for kidney failure incidence and, by extension, CKD incidence rates. As far as the authors are aware, there is no current CKD screening in Guatemala, including in the sugar cane plantations. As such, increased screening for CKD in Southwest Guatemala seems very unlikely. As renal replacement therapy is not widely available in most of Guatemala, we suspect that our data underestimate the true burden of kidney disease and failure.

## Conclusions

The overlap between dialysis enrollment rates, agricultural production (sugar cane), average daytime harvest temperatures, and the predominance of male enrollees supports the hypothesis that a similar disease pattern – likely CKDnT - is occurring in Southwest Guatemala as in western Nicaragua and El Salvador.

The etiology of and possible solutions to CKD in this region merits additional and urgent research.

## Abbreviations

CENCAM: Consorcio de la Epidemia de la Nefropatía en Centroamérica y México (Consortium for the Epidemic of Nephropathy in Central America and Mexico)
**C**engicaña: Centro Guatemalteco de Investigación y Capacitación de la Caña de Azúcar (Guatemala Sugar Cane Research and Training Center)
CISTA: Centro de Investigación en Salud, Trabajo y Ambiente (Center for Investigation in Health, Work and Environment)
CKD: Chronic Kidney Disease
CKDnT: Chronic Kidney Disease of Non-Traditional Causes
CKDu: Chronic Kidney Disease of Unknown Origins
HDI: Human Development Index
MeN: Mesoamerican Nephropathy
PD: Peritoneal Dialysis
r_s_: Spearman’s rank order correlation coefficient (Spearman’s rho)
UNAERC: Unidad Nacional de Atención al Enfermo Renal Crónico (The National Center for Chronic Kidney Disease Treatment)
UNAN-Leon: Universidad Nacional Autónoma de Nicaragua, León (National Autonomous University of Nicaragua at León)
